# Cardiac and plasma lipid profiles in response to acute hypoxia in neonatal and young adult rats

**DOI:** 10.1186/1476-511X-9-3

**Published:** 2010-01-13

**Authors:** Eric D Bruder, Hershel Raff

**Affiliations:** 1 Endocrine Research Laboratory, Aurora St. Luke's Medical Center, Milwaukee, WI 53215, USA; 2 Department of Medicine, Medical College of Wisconsin, Milwaukee, WI 53226, USA

## Abstract

**Background:**

The physiological and biochemical responses to acute hypoxia have not been fully characterized in neonates. Fatty acids and lipids play an important role in most aspects of cardiac function.

**Methods:**

We performed comprehensive lipid profiling analysis to survey the changes that occur in heart tissue and plasma of neonatal and young adult rats exposed to hypoxia for 2 h, and following 2 h of recovery from hypoxia.

**Results:**

Cardiac and plasma concentrations of short-chain acylcarnitines, and most plasma long-chain fatty acids, were decreased in hypoxic neonates. Following recovery from hypoxia, concentrations of propionylcarnitine, palmitoylcarnitine, stearoylcarnitine were increased in neonatal hearts, while oleylcarnitine and linoleylcarnitine concentrations were increased in neonatal plasma. The concentrations of long-chain fatty acids and long-chain acylcarnitines were increased in the hearts and plasma of hypoxic young adult rats; these metabolites returned to baseline values following recovery from hypoxia.

**Conclusion:**

There are differential effects of acute hypoxia on cardiac and plasma lipid profiles with maturation from the neonate to the young adult rat. Changes to neonatal cardiac and plasma lipid profiles during hypoxia likely allowed for greater metabolic and physiologic flexibility and increased chances for survival. Persistent alterations in the neonatal cardiac lipid profile following recovery from hypoxia may play a role in the development of rhythm disturbances.

## Background

Pulmonary and cardiac dysfunction results in hypoxemia in neonates that, in turn, may lead to hypothermia and bradycardia [1-5]. Myocardial lipid composition affects electrophysiological and mechanical function due to modulation of the physicochemical properties of cellular membranes [6-11]. Fatty acids also generate a constant supply of ATP needed for normal myocardial function [12]. Perturbations in cardiac fatty acid composition may lead to defects in ion channel function, decreases in mitochondrial oxidative capacity, and rhythm disturbances [13-16].

The present study evaluated cardiac and plasma lipid, fatty acid, and acylcarnitine profiles in response to and after recovery from severe hypoxia in neonatal (PD2) and young adult (PD60) rats. We identified hypoxia-induced changes in cardiac and plasma lipid profiles that may be important in cardiac function, either during or recovery from hypoxia.

## Methods

The Aurora Health Care IACUC approved the animal protocol. Timed-pregnant Sprague-Dawley rats at gestational day 15 (N = 6) and male rats at postnatal day (PD) 50 (175-200 g; N = 12) from Harlan Sprague Dawley (Indianapolis, IN) were maintained on a standard diet and water *ad libitum *in a controlled environment (0600-1800 lights on). The size of litters was normalized (12 pups/litter; N = 72 pups). PD2 pups were placed in chambers and allowed to nest and huddle on an adequate amount of bedding. After 30 min of room air (8 L/min), 3-4 pups from each litter were removed from the chamber, sacrificed, and baseline samples pooled. Then, the input O_2 _concentration was decreased to 8%. After 2 h, 3-4 pups from each litter were removed from the chamber, sacrificed, and samples pooled; the O_2 _concentration in each chamber was then returned to 21%. Remaining pups were sacrificed following a 2 h recovery period. PD60 rats were similarly exposed to hypoxia (3 rats per chamber).

Rats were sacrificed by decapitation and trunk blood was collected into EDTA. Blood samples were centrifuged for 10 sec (within 10 sec of collection) in a microfuge at room temperature with plasma frozen immediately. Plasma samples from PD2 pups were pooled (4 pups/sample). Whole hearts from PD2 rats were immediately excised, rinsed in ice-cold saline, and frozen in liquid nitrogen (3 @ PD2 hearts per sample). Plasma and hearts from each PD60 rat was treated as one sample (N = 4 sacrificed per time point).

A comprehensive assessment of heart and plasma lipid profiles was performed (Lipomics Technologies, Inc., West Sacramento, CA) as described previously [17]. Intra-assay CVs were: cholesterol ester (CE; 2.0%), diacylglyercol (DAG; 5.5%), free fatty acid (FFA; 3.5%), lysophosphatidylcholine (LPC; 12.2%), phosphatidylcholine (PC; 5.0%), phosphatidylethanolamine (PE; 13.0%), phosphatidylserine (PS; 10.0%) and triacylglycerol (TAG; 0.4%). Samples for acylcarnitine profiling were prepared for liquid chromatography-tandem mass spectrometry (LC-MS/MS) analysis in the presence of dueterated surrogates for quantitation through a modified liquid preparation and injected onto an 1100 Series HPLC (Agilent Technologies, CA) connected to a Quattro Premier triple quadrupole mass spectrometer (Waters, MA). The analytes were ionized via positive electrospray and the mass spectrometer was operated in the tandem MS mode.

Data are expressed as mean ± SEM. Significant differences were assessed by one-way ANOVA with post hoc Student-Newman-Keuls analysis for multiple comparisons (SigmaStat 2.03). Differences in baseline lipid class concentrations (PD2 vs. PD60) were assessed by unpaired Student's t-test.

## Results

Hearts from PD60 rats had higher baseline FFA, PC, and PE when compared to hearts from PD2 rats (Table 1). Plasma from PD60 rats had significantly higher CE at baseline, while DAG, PC, PE, and TAG were lower compared to plasma from PD2 rats.

**Table 1:** Baseline lipid class concentrations (nmol/g) in the heart and plasma of PD2 and PD60 rats - effects of age.

Lipid Class	Source	PD2	PD60	P Value
**Cholesterol Ester (CE)**	**Heart**	337.75 ± 96.09	424.50 ± 102.75	0.560
	**Plasma**	1249.00 ± 41.23	1449.00 ± 48.61*	0.020
				
**Diacylglycerol (DAG)**	**Heart**	293.75 ± 30.56	334.00 ± 13.10	0.272
	**Plasma**	65.75 ± 8.84	30.00 ± 7.22*	0.020
				
**Free Fatty Acid (FFA)**	**Heart**	1230.00 ± 113.48	2093.50 ± 153.14*	0.004
	**Plasma**	689.25 ± 53.22	653.00 ± 27.42	0.567
				
**Lysophosphatidylcholine (LPC)**	**Heart**	423.75 ± 35.01	487.00 ± 36.79	0.259
	**Plasma**	394.25 ± 13.92	393.00 ± 34.47	0.974
				
**Phosphatidylcholine (PC)**	**Heart**	8091.75 ± 759.67	14169.25 ± 789.30*	0.001
	**Plasma**	1246.25 ± 35.92	1130.50 ± 21.52*	0.033
				
**Phosphatidylethanolamine (PE)**	**Heart**	6896.00 ± 624.50	12343.00 ± 434.87*	<0.001
	**Plasma**	324.00 ± 23.01	181.75 ± 24.55*	0.006
				
**Phosphatidylserine (PS)**	**Heart**	1321.33 ± 159.17	1614.75 ± 62.90	0.112
	**Plasma**	n/a	n/a	
				
**Triacylglycerol (TAG)**	**Heart**	1038.00 ± 147.45	999.25 ± 360.61	0.924
	**Plasma**	1527.50 ± 108.83	331.50 ± 39.01*	<0.001
				
**Free Cholesterol (FC)**	**Heart**	3762.25 ± 327.30	3651.00 ± 151.38	0.768
	**Plasma**	632.75 ± 30.51	622.25 ± 22.22	0.790

Data are presented as mean ± SEM. * Significantly different from PD2 rats. N = 4 samples for each experimental group. Note: plasma from 2-3 pups was pooled to generate one sample. N/A = not applicable.

Table 2 summarizes only those alterations that had statistically significant cardiac lipid profiles. PD2 cardiac TAG-20:5n3 was decreased, while PE-14:0, PS-18:2n6, and PS-20:4n6 were increased by exposure to hypoxia. Following recovery from hypoxia, PD2 cardiac DAG-16:0, DAG-18:0, PC-18:2n6, PS-dm16:0, and PC-20:4n6 were increased. Additionally, PS-18:2n6 and PS-20:4n6 remained significantly increased following recovery from hypoxia when compared to baseline. In the hearts of PD60 rats, 16:0, 16:1n7, 18:1n9, 18:3n3, and 18:2n6 in the FFA fraction were increased by exposure to acute hypoxia. There were no significant changes in the PD60 cardiac lipid profile following recovery from hypoxia.

**Table 2:** Cardiac lipid profiling in PD2 and PD60 rats - effects of hypoxia and recovery.

Lipid Class - Fatty Acid	Baseline	2 h of Hypoxia	2 h Recovery From Hypoxia
**PD2 Heart**			
			
**PE - 14:0**	18.12 ± 1.94	33.72 ± 3.22 b	25.15 ± 3.88
**TAG - 20:5n3**	23.40 ± 2.23	13.97 ± 1.50^a^	18.22 ± 2.15
**DAG - 16:0**	162.65 ± 14.40	181.48 ± 10.22	215.03 ± 13.13^a^
**DAG - 18:0**	73.10 ± 5.66	79.82 ± 4.74	94.55 ± 2.95^a^
**PC - 18:2n6**	910.23 ± 51.81	1016.28 ± 43.05	1137.85 ± 37.57^b^
**PC - 20:4n6**	3357.63 ± 241.70	3829.30 ± 187.24	4163.98 ± 91.42^a^
**PE - 22:5n3**	348.03 ± 31.18	376.00 ± 13.45	445.35 ± 21.60^a^
**PS - 18:2n6**	73.93 ± 3.22	84.22 ± 3.55^a^	88.72 ± 1.69^b^
**PS - 20:4n6**	249.07 ± 29.52	317.28 ± 15.47^a^	368.38 ± 5.09^d^
**PS - dm16:0**	29.67 ± 3.93	41.53 ± 3.45	45.65 ± 3.74^a^
			
**PD60 Heart**			
			
**FFA - 16:0**	503.55 ± 32.46	715.00 ± 70.41^a^	539.00 ± 18.51
**FFA - 16:1n7**	12.15 ± 0.55	44.85 ± 7.77^d^	15.00 ± 1.07
**FFA - 18:1n9**	200.07 ± 15.70	420.65 ± 49.30^d^	281.20 ± 11.50
**FFA - 18:3n3**	15.47 ± 1.26	37.97 ± 3.96^e^	18.77 ± 1.39
**FFA - 18:2n6**	438.52 ± 39.99	710.65 ± 90.36^a^	546.32 ± 32.84
**FFA (total)**	2093.50 ± 153.14	3000.00 ± 303.04^a^	2375.00 ± 88.179
**TAG - 20:4n6**	148.50 ± 21.07	324.10 ± 51.47^a^	182.02 ± 43.99
**TAG - 18:1n9**	517.25 ± 241.95	2694.30 ± 902.49^a^	500.62 ± 96.65
**TAG (total)**	999.25 ± 360.61	3986.00 ± 1295.06^a^	962.25 ± 163.08

Animals were exposed to hypoxia (8% O_2_) for 2 h or exposed to hypoxia for 2 h and allowed to recover in room air (21% O_2_) for an additional 2 h. Due to the size of the data set produced by the lipid profiling analyses, only significant changes (p < 0.05) are shown. Data are presented as mean ± SEM (nmol/g tissue). All comparisons were made versus Baseline as follows: ^a ^P < 0.05, ^b ^P < 0.02, ^c ^P < 0.01, ^d ^P < 0.005, and ^e ^P < 0.001. Lipid class abbreviations can be found in the legend of Table 1.

Total plasma FFA concentrations, including 16:0, 18:1n9, and 18:2n6, were significantly decreased by exposure to hypoxia in PD2 rats (Table 3); there were no changes following recovery. Plasma total FFA (e.g. 16:0, 16:1n7, 18:1n9, and 18:2n6) was increased by exposure to hypoxia in PD60 rats. Plasma TAG-20:4n6 and LPC-20:4n6 were altered by hypoxia, and these alterations remained following recovery from hypoxia.

**Table 3:** Plasma lipid profiling in PD2 and PD60 rats - effects of hypoxia and recovery.

Lipid Class - Fatty Acid	Baseline	2 h of Hypoxia	2 h Recovery From Hypoxia
**PD2 Plasma**			
			
**FFA - 16:0**	173.62 ± 15.61	90.62 ± 5.42^e^	146.32 ± 7.90
**FFA - 18:1n9**	80.02 ± 10.08	37.60 ± 2.89^d^	66.52 ± 3.73
**FFA - 18:2n6**	104.57 ± 6.31	47.90 ± 5.80^e^	91.22 ± 3.16
**FFA (total)**	689.25 ± 53.22	344.50 ± 26.29^e^	614.75 ± 27.21
			
**PD60 Plasma**			
			
**FFA - 16:0**	192.52 ± 10.04	350.65 ± 12.19^e^	160.42 ± 14.72
**FFA - 16:1n7**	19.45 ± 1.24	50.07 ± 2.90^e^	17.00 ± 2.24
**FFA - 18:1n9**	101.42 ± 5.50	211.85 ± 3.29^e^	82.70 ± 8.92
**FFA - 18:2n6**	167.85 ± 3.99	363.10 ± 11.27^e^	134.65 ± 18.16
**FFA (total)**	653.00 ± 27.42	1249.00 ± 46.82^e^	538.00 ± 53.79
**TAG - 20:4n6**	76.10 ± 8.94	177.45 ± 24.25^d^	128.22 ± 9.35^a^
**LPC - 20:4n6**	54.57 ± 5.55	36.55 ± 5.81^a^	34.35 ± 1.46^a^

Animals were exposed to hypoxia (8% O_2_) for 2 h or exposed to hypoxia for 2 h and allowed to recover in room air (21% O_2_) for an additional 2 h. Due to the size of the data set produced by the lipid profiling analyses, only significant changes (p < 0.05) are shown. Data are presented as mean ± SEM (nmol/g plasma). All comparisons were made versus Baseline as follows: ^a ^P < 0.05, ^b ^P < 0.02, ^c ^P < 0.01, ^d ^P < 0.005, and ^e ^P < 0.001. N = 4 samples for each experimental group. Note: plasma from 2-3 PD2 rats was pooled to generate one sample. Lipid class abbreviations can be found in the legend of Table 1.

**Figure 1:**
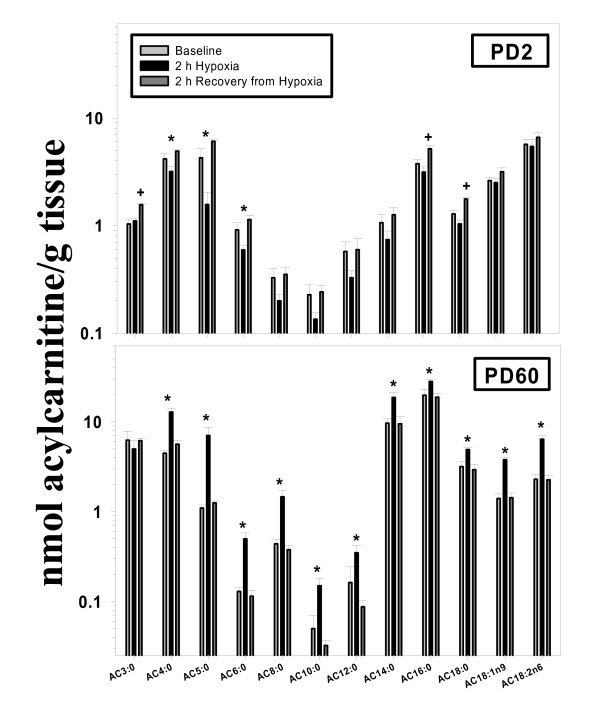
**Effects of exposure to and recovery from acute hypoxia in the PD2 (upper panel) and PD60 (lower panel) rat: cardiac acylcarnitine profiling**. Rats were sacrificed at baseline (black bars), 2 h after the onset of hypoxia (light gray bars), or 2 h after return to normoxia (dark gray bars). Acylcarnitine molecular species are listed on the x-axis. N = 4 per time point (within each age group). *Significant difference from baseline by one-way ANOVA (P < 0.05). ^+^Significant difference from baseline and 2 h of hypoxia by one-way ANOVA (P < 0.05).

Cardiac acylcarnities AC4:0, AC5:0, and AC6:0 were decreased during hypoxia in PD2 rats (Figure 1). After recovery, these metabolites returned to baseline, but AC3:0, AC16:0, and AC18:0 were increased. Interestingly, most AC metabolites were increased by exposure to hypoxia in PD60 hearts; these changes returned to baseline values following recovery. Carnitine concentrations were significantly lower in PD2 versus PD60 hearts; however, there was no effect of hypoxia or recovery in either age group.

**Figure 2:**
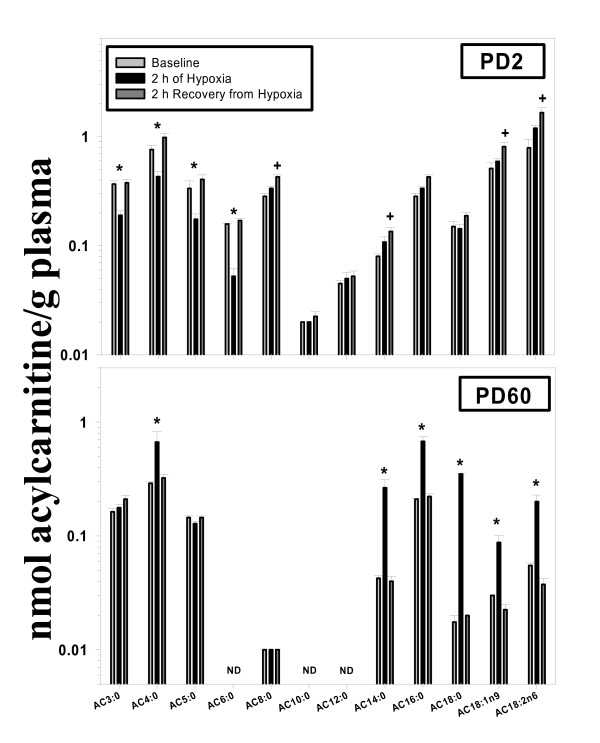
**Effects of exposure to and recovery from acute hypoxia in the PD2 (upper panel) and PD60 (lower panel) rat: plasma acylcarnitine profiling**. Rats were sacrificed at baseline (black bars), 2 h after the onset of hypoxia (light gray bars), or 2 h after return to normoxia (dark gray bars). Acylcarnitine molecular species are listed on the x-axis. N = 4 per time point (within each age group). Plasma samples for PD2 rats were pooled in order to generate one sample (N = 4 pups/sample). *Significant difference from baseline by one-way ANOVA (P < 0.05). ^+^Significant difference from baseline and 2 h of hypoxia by one-way ANOVA (P < 0.05). ND = not detected.

Plasma AC3:0, AC4:0, AC5:0, and AC6:0 were significantly decreased during hypoxia in PD2 rats (Figure 2). Following recovery, PD2 plasma AC8:0, AC14:0, AC18:1n9, and AC18:n6 were significantly increased. AC4:0, AC14:0, AC16:0, AC18:0, AC18:1n9, and AC18:2n6 were all increased by exposure to hypoxia in PD60 rats. Plasma carnitine concentrations were significantly higher in PD2 rats when compared to PD60 rats (P = 0.017), but hypoxia had no effect at both ages.

## Discussion

This comprehensive survey generated a large data set, yet a limited number of statistically significant changes were identified, demonstrating the specificity of the analysis. We will focus only on a subset of these changes and propose importance for their effects on cardiac function during or after exposure to hypoxia.

Hearts from PD2 rats exhibited resistance to hypoxia-induced changes in the lipid profile. Hypoxia caused a significant decrease in short-chain AC in the PD2 heart. This may have been due to increased mitochondrial β-oxidation under hypoxic conditions, as decreases in AC suggest their disappearance into mitochondria for subsequent metabolism. Conversely, PD2 hearts may have reverted to the oxidation of glucose (i.e. the fetal phenotype) under hypoxic conditions [18].

Following recovery from hypoxia, hearts from PD2 rats showed increases in 18:2n6, 20:4n6, and dm16:0 in the PS fraction. An increase in cardiac PS-associated n6 fatty acids and plasmalogen (i.e. dm16:0) may have altered the fluidity of cellular membranes, affecting membrane-associated signal transduction and intracellular Ca^2+ ^dynamics [19,20]. AC16:0 and AC18:0 were also increased in PD2 hearts following recovery from hypoxia. These long-chain AC's promote arrhythmogenesis via disruption of ion transport in sarcolemmal membranes and also induce mitochondrial and cellular uncoupling [21,22], suggesting that the PD2 heart may be at risk for rhythm disturbances following recovery from acute hypoxia.

As opposed to the PD2 heart, the lipid and fatty acid profile of the PD60 heart exhibited many changes in response to acute hypoxia. The FFAs 16:0, 16:1n7, 18:1n9, 18:3n3, and 18:2n6, and all long-chain AC metabolites measured, were increased by hypoxia. Accumulation of long-chain FFA and their AC counterparts have been shown to be detrimental to cardiac function [22-24]. Remarkably, following recovery from acute hypoxia, the lipid and fatty acid profiles of the PD60 heart were not different from baseline values.

All major plasma FFA were significantly decreased by exposure to hypoxia in PD2 pups. The disappearance of plasma FFA may have been due to increased insulin concentrations, and could prove beneficial in the response to hypoxia. For example, enhanced FFA utilization for hepatic gluconeogenesis may have been cardioprotective by providing the glucose needed for myocardial ATP generation [25]. Following recovery from hypoxia, the concentrations of AC18:1n9 and AC18:2n6 were significantly elevated. Unsaturated AC metabolites may lead to rhythm disturbances in the heart, as has been shown in myocardial ischemia [26].

Exposure of PD60 rats to hypoxia elicited significant increases in plasma metabolites from most fatty acid families in the FFA fraction. Plasma FFA and long-chain AC accumulation is often found in patients with acute myocardial infarction, and may negatively affect cardiac function through a number of inter- and intracellular mechanisms [15,21,22,24,27]. Plasma TAG-20:4n6 was increased in PD60 rats exposed to hypoxia and remained increased following recovery, and may cause contractile dysfunction and intracellular calcium overload [28]. Furthermore, increases in plasma lipid and fatty acids coincided with similar elevations of the same lipid metabolites in heart tissue.

The significant changes observed in this robust data set should serve as a basis for further exploration of the effects of acute hypoxia in the developing heart. The study highlighted changes in lipid metabolism that could potentially affect cardiac function, either in a positive or negative manner. These comprehensive analyses should lead investigators to focus on specific pathways relevant to the changes in lipid metabolites we have highlighted.

## Competing interests

The authors declare that they have no competing interests.

## Authors' contributions

EB carried out the animal experiments, analysis of the data, statistics, wrote the first draft, and finalized the manuscript. HR conceived the study, helped in the analysis of data and statistics, and the edited the subsequent drafts. Both authors read and approved the final manuscript.
